# Fit for Cancer Treatment: a prospective feasibility study of primary care initiated prehabilitation for patients with suspected cancer

**DOI:** 10.3399/bjgpopen18X101608

**Published:** 2018-10-17

**Authors:** Rachael C Barlow, David Sheng Yi Chan, Sharon Mayor, Ceri Perkins, Helen L Lawton, Arfon GMT Powell, Wyn G Lewis

**Affiliations:** 1 Lecturer, College of Biomedical and Life Science, Cardiff University, Cardiff, UK; 2 Clinical Academic, Department of Surgery, Cardiff and Vale University Health Board, Cardiff, UK; 3 ST8 in General Surgery, Department of Surgery, Cardiff and Vale University Health Board, Cardiff, UK; 4 Senior Lecturer, College of Biomedical and Life Science, Cardiff University, Cardiff, UK; 5 Research Coordinator, Department of Surgery, Cardiff and Vale University Health Board, Cardiff, UK; 6 GP, Radyr Medical Centre, Cardiff, UK; 7 WCAT Lecturer in Surgery, Division of Cancer and Genetics, Cardiff University School of Medicine, Cardiff, UK; 8 Consultant Surgeon and Head of Specialty School Surgery, Wales Post Graduate Medical and Dental Education Deanery School of Surgery, Cardiff University, Cardiff, UK

**Keywords:** prehabilitation, primary care, urgent suspected cancer, general practice, cancer, neoplasms

## Abstract

**Background:**

Risk profile assessment and corrective interventions using optimisation of health status and prehabilitation represent an important strategy in the management of patients with a suspected cancer diagnosis.

**Aim:**

To determine the feasibility of pre-treatment optimisation and prehabilitation commenced at index primary care consultation, to improve patients’ preparation for treatment by maximising the time available.

**Design & setting:**

Between January 2015 and May 2016, 195 patients presenting to 12 GP practices were deemed eligible to enter the study, of which 189 (96.9%, median age 60 [21–91] years and 65 months; 124 female) were recruited and consented to the prehabilitation bundle.

**Method:**

All patients were simultaneously referred to secondary care using urgent suspected cancer (USC) pathways. The primary outcome measures were definitive diagnosis and treatment plan.

**Results:**

Fifteen patients (7.9%) were diagnosed with cancer (three breast, three colon, two lung, two skin [one melanoma, one sarcoma], one tonsil, one vocal cord, one pancreas, one prostate, one ependymoma) and 62 were diagnosed with other significant medical conditions (47 gastrointestinal, 13 sepsis, two respiratory) requiring secondary care assessment and treatment. Of the 15 patients with cancer, 11 (73.3%) underwent potentially curative treatment, and four (26.7%) palliative treatment. Of the total study cohort, 84 (44%) required a form of optimisation in primary care, and patients with cancer were more likely to require optimisation than others (*n* = 10 [63%] versus *n* = 74 [43%], χ^2^ 9.384, *P* = 0.002).

**Conclusion:**

One in 12 primary care USC patients had cancer (5.6% receiving potentially curative treatment), one in three had other systemic health issues, and overall two in five benefited from healthcare intervention. Primary care optimisation was feasible and associated with important allied health benefits.

## How this fits in

Previous prehabilitation studies have focused on hospital secondary care interventions rather than action started at the index primary care consultation when the USC referral is made.

This study assesses the feasibility of a pre-treatment optimisation and prehabilitation bundle commenced in primary care. This is important to maximise the time available for remedial interventions to take effect, should the patient require any treatments at a later date. 

One in 12 primary care USC patients had a cancer diagnosis confirmed (5.6% receiving potentially curative treatment), one in three were diagnosed with systemic health issues, and overall two in five were likely to benefit from healthcare intervention.

A primary care optimisation strategy was feasible and associated with important allied health benefits.

## Introduction

Suspicion of cancer drives urgent action in the modern NHS, yet threats to successful treatment are common and multifactorial, and risk profile assessment is critical to enhancing clinical outcomes. The concept of prehabilitation refers to an emerging field of research concerned with strategies to optimise patients’ pre-treatment physiological, functional, and psychosocial risk profiles, such that post-treatment recovery trajectories are boosted. This results in fewer complications, shorter durations of hospital stay, improved quality of life, better survival, and prudent, cost-effective health care.^1,2^ Reports to date have focused on a heterogeneous collection of health interventions, delivered within the care continuum, and conveyed between diagnosis and treatment. Education, exercise, nutrition, and psychosocial approaches have all been included, focused not only on the patient, but also the patient’s family, aimed at promoting health-related behavioural change, reaching beyond the pre-treatment interval into the long-term future.^3^

To date, research has focused on hospital secondary care interventions, rather than action started at the index primary care consultation, when the USC referral is made. USC referrals receive prioritised action by hospital cancer networks, yet at least 2 weeks may pass before diagnostic investigations occur, and further delays may occur as results are processed. Moreover, cancer site-specific staging protocols can be complex, involving both radiological and physiological staging investigations, building further delays into patients’ cancer journeys and treatment pathways, as described by the Aarhus statement as the system interval.^4^

The aim of this study was to investigate whether primary care optimisation and prehabilitation was feasible and logistically reliable in the face of a USC referral. Secondary objectives included clinical application and compliance. Primary outcome measures were definitive diagnosis, treatment plan, time to treatment, and treatment-associated complication rates. The hypothesis was that optimisation and prehabilitation commenced in primary care was feasible, and would optimise use of the combined doctor and system interval between patients’ clinical appearance and treatment. The setting consisted of 12 primary care GP practices in Cardiff, UK, which has 80 general practices, serving a population of 350 000.

## Method

**Figure 1.**
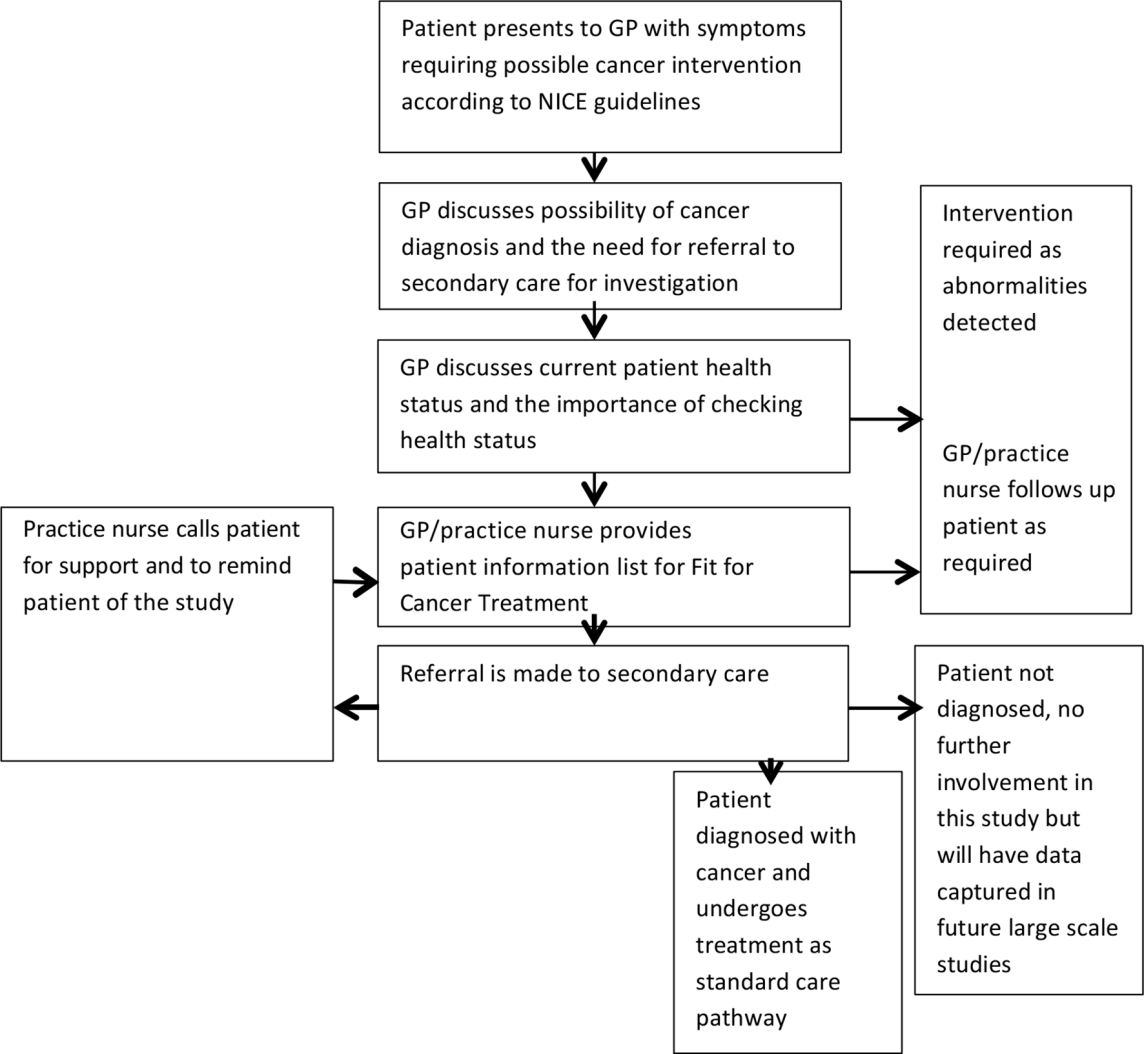
Patient flow diagram

The study was a prospective, interventional feasibility trial and, to minimise bias, the team collecting patient data had no direct involvement with patient care. Twelve GP primary care practices within the catchment population of Cardiff and Vale University Health Board were employed. Patients presenting with symptoms compatible with a suspected diagnosis of primary cancer between 1 January 2015 and 31 May 2016 were eligible to enter the study. Inclusion criteria demanded that all patients were aged ≥18 years, and were simultaneously referred to secondary care via USC pathways (Figure 1). Sample size was calculated in order to enable a pragmatic estimation with regard to the factors of interest, in particular variability, without exposing excess numbers of patients to the full package of health interventions. Based on the local audited conversion rate of USC referral to cancer diagnosis of 8%, it was anticipated that recruitment of 200 patients would be required in order to result in a cohort of 20 patients with a definitive cancer diagnosis after secondary care referral. Primary outcome measures were definitive diagnosis, treatment plan, time to treatment, and treatment-associated complication rates.

Recruitment commenced 1 January 2015 and closed 31 May 2016, with patient follow-up completed on 30 November 2016.

### Recruitment

Potential participants were identified at their initial GP consultation when presenting with symptoms suspicious of primary cancer. At the index appointment, GPs discussed the need for referral to secondary care for investigation and the possibility of a cancer diagnosis was shared. In addition, potential participants were asked if they were content to receive information regarding the study and, if they agreed, an information leaflet was provided. Patients were given at least 48 hours after this consultation before further contact with the GP or primary care practice nurse to enquire how they were coping with events and potential diagnosis. Patients recruited to the study were invited to a face-to-face appointment with either the primary care or research nurse, if available in the practice, to discuss the trial in more detail and informed consent was obtained.

### Proposed intervention

**Figure 2.**
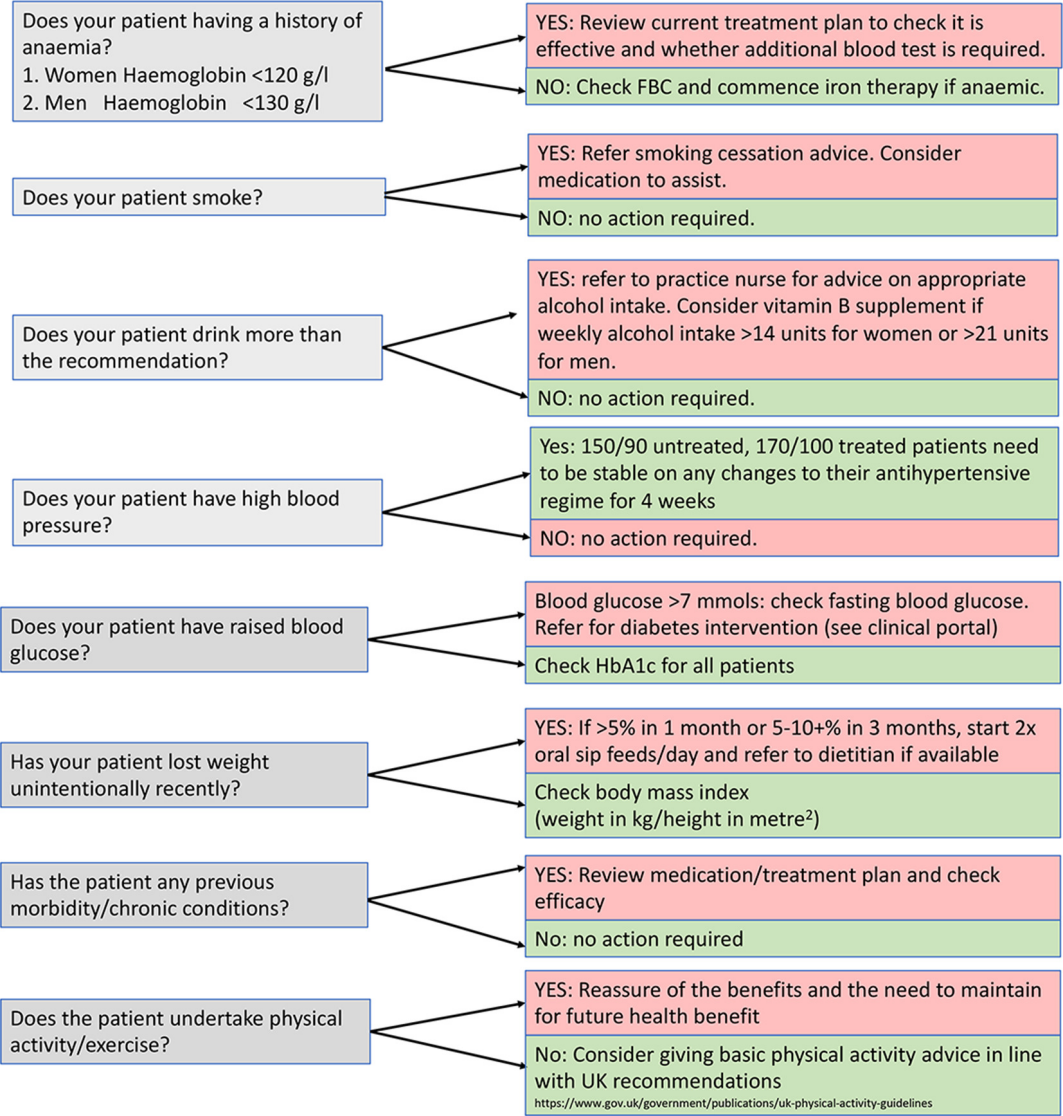
Optimisation bundle assessment FBC = full blood count. HbA1C = haemoglobin A1c.

**Figure 3.**
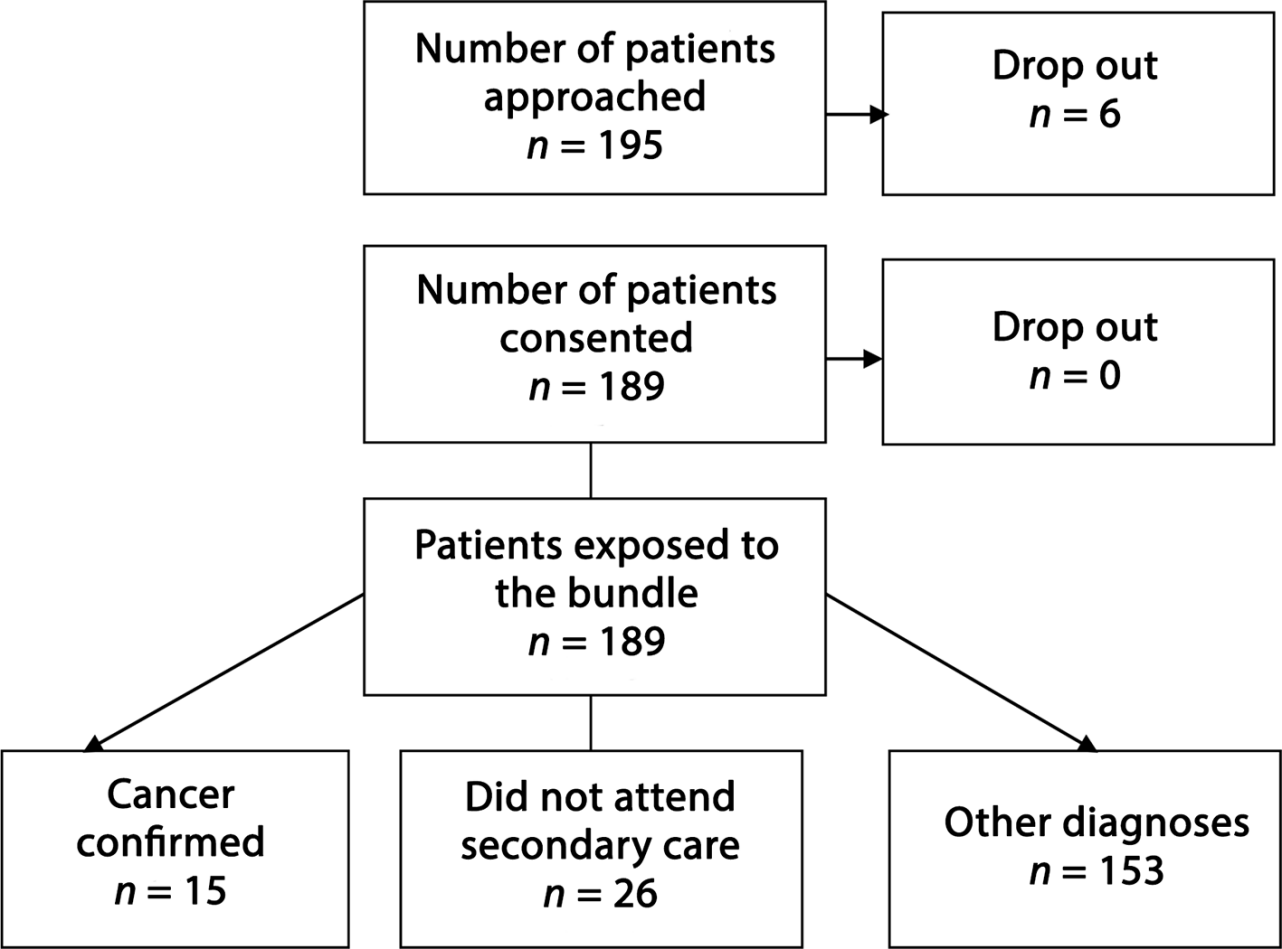
Flow diagram outlining details of patients who were recruited into study

Each participating general practice received support and training by a member of the research team in keeping with principles of good clinical practice. After obtaining informed written consent, patients underwent an optimisation and prehabilitation bundle assessment (Figure 2) with the aid of a template developed using the Vision primary care database or EMIS Health systems. The key components of the bundle are illustrated in Figure 3, which was finalised with the aid of local focus groups of health professionals residing in the Cardiff and Vale University Health Board geographical area.

### Data collection and statistical analysis

Data were recorded in paper-case report forms at the time of each patient contact and anonymised. This information was supplemented by the Vision or EMIS patient record system. Statistical analysis appropriate for non-parametric data (Kruskal-Wallis and Mann-Whitney U-tests) was performed using SPSS (version 23).

## Results

During the study period, 195 patients were approached for enrolment, of which six declined, resulting in a total recruitment cohort of 189 patients, all of whom received the optimisation bundles in primary care within the allotted time. The median age of the participants was 60 (range 21–91) years and the sex ratio was 65 (34.4%) male to 124 (65.6%) female. Fifteen patients were diagnosed with cancer (nine male, six female, median age 59 [42–84] years). The cancer specific sites were: breast (*n* = 3), colon (*n* = 3), lung (*n* = 2), skin (*n* = 2 [one melanoma, one sarcoma]), tonsil (*n* = 1), vocal cord (*n* = 1), pancreas (*n* = 1), prostate (*n* = 1), and ependymoma (*n* = 1), as detailed in Table 1. Eleven patients (73.3%) were treated with curative intent. The median time from diagnosis to treatment was 29 days (range 1–76). Twenty-six patients failed to attend the secondary care referral appointments, and therefore 148 patients were subsequently confirmed to have non-cancer related diagnoses in secondary care (62 patients were diagnosed with other significant medical conditions: 47 gastrointestinal, 13 sepsis, and two respiratory). Eighty-four patients (44.4%) in total required some form of remedial intervention or optimisation at the initial primary care appointment. The proportion of patients diagnosed with cancer requiring intervention or optimisation was significantly higher than that of the patients with other diagnoses (*n *= 10 [66.6%] versus 74 [42.5%], χ^2^ 9.384, 1 degree of freedom 1, *P* = 0.002).

**Table 1.** Details of patients diagnosed with cancer

**Patient code**	**Age**	Sex	**Cancer**	**Stage**	**Treatment**	**Complication**
1	57	Female	Breast	III	Surgery	None
2	52	Male	Tonsillar	II	Chemoradiotherapy	None
3	81	Male	Lung	IV	Palliative	
4	59	Male	Lung	IV	Palliative	
5	54	Male	Vocal cord	II	Radiotherapy	None
6	53	Male	Prostate	I	Surveillance	
7	55	Female	Pancreas	IV	Palliative	
8	42	Male	Schwannoma	I	Surgery	None
9	57	Female	Breast	III	Surgery	None
10	63	Female	Breast	II	Surgery	Seroma
11	82	Male	Melanoma	II	Surgery	None
12	65	Female	Rectal	I	Polypectomy	None
13	84	Male	Rectal	IV	Palliative	
14	67	Male	Colon	II	Surgery	None
15	64	Female	Ependymoma	I	Surgery	None

### Reasons for uptake of optimisation bundle

Of the total study cohort, 84 patients (44.4%) were detected as requiring optimisation in primary care. Of the total study cohort, 84 patients (44.4%) were detected in primary care to require optimisation. Of these 84 patients who required intervention, lifestyle advice relating to physical activity, diet, and alcohol accounted for the majority. Of the total study cohort, 87 patients were already on the hypertension register with an additional four patients detected and started on appropriate interventions as a result of this study; none of these four patients went on to have a cancer diagnosis. Diabetes mellitus pre-existed in 26 patients (13.8%) and 19 were found to have elevated blood glucose. Exercise levels varied widely: 25 patients (13.2%) were exercising for >120 minutes per week and none were diagnosed with cancer; 45 patients took no exercise at all, and proportionately more received cancer diagnoses (*n* = 6 [50%] versus *n* = 39 [25%]), as shown in Table 2. Exercise levels were not recorded in 10 patients. Nutrition advice was needed in 30 patients (15.9%); three were signposted to a dietician for counselling (Table 3). Thirty-two patients (16.9%) had a previous diagnosis of anaemia, nine of whom (28.1%) were not receiving iron therapy. Anaemia was detected in 19 patients (10.1%) and all commenced remedial iron therapy. In the cancer cohort, six patients (40.0%) had either pre-existing or newly detected anaemia.

**Table 2.** Details of levels of exercise related to diagnosis

	**Cancer, *n* (%)**	**Other, *n* (%)**
Total, *n*	15	154
No exercise	6 (40.0)	39 (25.3)
>30 mins	5 (33.3)	60 (38.9)
>60 mins	1 (6.7)	19 (12.3)
>90 mins	0 (0.0)	3 (1.9)
>120 mins	0 (0.0)	25 (16.2)
Not recorded	3 (20.0)	7 (4.5)

**Table 3.** Details of nutrition related to diagnosis

	**Cancer, *n* (%)**	**Other, *n* (%)**
Total, *n*	15	154
Low BMI	2 (13.3)	11 (7.1)
Normal BMI	4 (41.6)	39 (25.3)
Overweight	5 (33.3)	48 (31.2)
Obese	5 (33.3)	56 (36.3)
Weight loss (0%)	7 (46.7)	130 (84.4)
Weight loss (>5%)	6 (40.0)	14 (9.1)
Weight loss (>10%)	3 (20.0)	6 (3.9)
Weight loss (>20%)	0 (0.0)	1 (0.6)

## Discussion

### Summary

The principal finding of this study was that primary care optimisation was feasible and associated with important potential allied health benefits, satisfying the hypothesis. One in 12 primary care USC patients had a cancer diagnosis confirmed (5.6% receiving potentially curative treatment), one in three were diagnosed with systemic health issues, and, overall, two in five were likely to benefit from healthcare intervention in primary care. Of the 15 patients diagnosed with cancer, eight (53.3%) underwent potentially curative surgery, of whom seven (87.5%) required optimisation. All but one of the patients diagnosed with tumours received treatment within 60 days of diagnosis (the patient with ependymoma was the exception), none had their treatment delayed for medical reasons, and none suffered significant therapy-related complications.

### Strengths and limitations

The study has a number of inherent potential limitations. The number of new cancers diagnosed was relatively modest, although in keeping with the audited USC conversion rate within the local health board. The cancer sites diagnosed were very diverse and assessing the impact of prehabilitation on treatment delay and outcome must consequently be performed with caution. The costs of developing this intervention on a wider scale may be significant; a 6% pick-up rate of potentially curable cancers implies that to effect potential beneficial change on 100 patients with cancer, approximately 1600 USC patients would need to receive the intervention. The study was not comparative, and no retrospective analysis was possible regarding the other patients diagnosed with cancer who may have been missed by the recruitment strategy. The allied healthcare benefits for the patients without a cancer diagnosis were not measured and quantified as this was outside the scope and funding of the project. In contrast, the strengths of the study lie in its originality, and the move in timing and healthcare level of the intervention. Moreover, the interventions described are relatively simple, feasible, and of general interest, and there were significant potential health-related benefits for patients without cancer; the economic benefits of this finding merit targeted examination and research.

### Comparison with existing literature

Surgical outcomes, in broad terms in the UK, have never been better or more transparent, despite the contemporary challenges of an ageing, sometimes frail population with increasing comorbidity.^5^ High-risk surgical patients are at greater danger of postoperative complications, prolonged durations of hospital stay, and blighted recovery because of compromised quality of life. Moreover, patients diagnosed with cancer face and pose specific problems, including debility, weight loss, malnutrition, and anaemia, which all influence surgical outcome. The addition of multimodal neoadjuvant therapies may be associated with survival benefit, but are often bought at a cost to physical function, in particular cardiorespiratory fitness and reserve when at a premium and most needed. Recent improvements in postoperative outcomes can be attributed to centralisation of cancer surgery, introduction of minimally invasive techniques, and enhanced recovery after surgery programmes (ERPs). ERPs are well established in many, but not all, surgical specialties, and have been reported widely to improve postoperative outcomes, yet most ERP elements focus on the immediate pre-, intra-, and postoperative phases of a patient’s hospital stay. Prehabilitation, in contrast, is founded on the principle that structured and coordinated preparation, including exercise in the preoperative period, provides patients with a psychological and physiological buffer to withstand surgical stress, which may carry the potential to minimise functional decline, reduce operative morbidity, and improve quality of life during convalescence.^6^

Hijazi,^7^ in a recent systematic review of prehabilitation programmes in abdominal cancer surgery, described nine studies: seven randomised and two prospective non-randomised trials, comprising 549 patients, 281 prehabilitation versus 268 controls. No significant difference was found between prehabilitation and control patients, although only five studies assessed postoperative morbidity. It was concluded that the programmes were heterogenous in composition, mode of administration, outcome measures of functional capacity, and all aspects were in need of standardisation prior to a larger scale evaluation.^7^ Arguably, the principal limitations of the above studies were that no fewer than seven of the prehabilitation programmes reported were unimodal rather than multimodal, focusing only on exercise intervention, ignoring the important lifestyle, nutritional, psychosocial, and general health status components, the significance of which should not be underestimated in patients with cancer.^8,9^ The current study was multimodal and included a generic health assessment optimisation bundle, screening on the one hand for pre-existing past medical history, and on the other hand for new health diagnoses, such as hyperglycaemia,^10^ anaemia,^11,12^ and hypertension,^13^ all of which are associated with poorer cancer treatment outcomes, especially surgery. Moreover, early detection of all of the above, allied to nutritional, exercise, and lifestyle advice, prolongs the remedial time available for intervention and potential benefit.

### Implications for research and practice

The character of cancer control is changing, with public opinion focusing on prevention, early diagnosis, and patient experience, before, during, and after treatment.^14^ Allied to this transformation, primary care is progressively promoted by health funders worldwide, as the favoured setting for most health care for reasons of need, cost, satisfaction of patient preference for care close to home. Consequently, it is opportune now to consider how a growing role for primary care can facilitate cancer treatment, which has long been dominated by highly technical interventions centred on treatment, and in which the input of primary care has been supposed to be largely marginal. Primary care’s strengths — synchronised, constant, and complete care for people and relations — are above all manifest in prevention and diagnosis, in shared follow-up and survivorship care, where a strong theme of integrated care exists. New models of care, including prehabilitation, present an opportunity to seize the so-called ‘teachable moment’, when the importance of positive lifestyle change can be stressed. Future research should explore the catalytic effect of combining prehabilitation with ERPs to quantify the potential surgical benefits, including cost-effectiveness evaluations. For patients undergoing oncological or palliative care the effect of prehabilitation on overall survival warrants attention. Finally, and by tradition, prehabilitation programmes are prescriptive and generic; employing a one-size-fits-all philosophy. Bespoke personalised programmes, which relate to individual patients' physiological, functional, and psychosocial needs, combining supervised and self-management interventions delivered in the community rather than secondary care, are likely to be associated with best compliance and effect.

## Trial registration

The study was registered with the UK Clinical Research Network (CRN) clinical trials website (UKCRN ID 16916).
